# Structural Basis of the Transcriptional Elongation Factor Paf1 Core Complex from *Saccharomyces eubayanus*

**DOI:** 10.3390/ijms24108730

**Published:** 2023-05-13

**Authors:** Yan Qin, Yuqiao Zhou, Yinghua Cao, Yanpeng Ren, Pujuan Deng, Junyi Jiang, Zhanxin Wang

**Affiliations:** Key Laboratory of Cell Proliferation and Regulation Biology of Ministry of Education, College of Life Sciences, Beijing Normal University, 19 Xinjiekouwai Avenue, Beijing 100875, China

**Keywords:** Paf1 complex, Rtf1, transcription elongation, evolution, crystal structure

## Abstract

The multicomponent polymerase associated factor 1 (Paf1) complex (PAF1C) is an important transcription elongation factor that upregulates RNA polymerase II-mediated genome-wide transcription. PAF1C can regulate transcription through direct association with the polymerase or by impacting the chromatin structure epigenetically. In recent years, significant progress has been made in understanding the molecular mechanisms of PAF1C. However, high-resolution structures that can clarify the interaction details among the components of the complex are still needed. In this study, we evaluated the structural core of the yeast PAF1C containing the four components Ctr9, Paf1, Cdc73 and Rtf1 at high resolution. We observed the interaction details among these components. In particular, we identified a new binding surface of Rtf1 on PAF1C and found that the C-terminal sequence of Rtf1 dramatically changed during evolution, which may account for its different binding affinities to PAF1C among species. Our work presents a precise model of PAF1C, which will facilitate our understanding of the molecular mechanism and the in vivo function of the yeast PAF1C.

## 1. Introduction

Most eukaryotic protein-coding genes and a large number of noncoding RNAs are transcribed by RNA polymerase II (Pol II). In prokaryotes, transcription initiation is the major stage of transcriptional regulation, while in eukaryotes, regulation at the elongation stage is also crucial for the expression of most Pol II-targeted genes. In metazoans, promoter-proximal pausing by Pol II is found at the majority of actively transcribed genes [1,2]. The paused Pol II needs the help of transcription elongation factors to be released from the paused state to complete the transcription process. To date, quite a few transcription elongation factors have been shown to function in the pause-release regulation of Pol II [1,2]. The polymerase-associated factor 1 complex (PAF1C) is one of the most important transcription elongation factors that positively regulates genome-wide gene expression [3].

PAF1C is a eukaryote-specific transcription elongation factor. In simple eukaryotes such as budding yeast, PAF1C contains five subunits: Paf1, Ctr9, Cdc73, Leo1 and Rtf1. In higher eukaryotes, such as humans, the PAF1 complex contains a sixth subunit, SKI8. A major function of PAF1C is to positively regulate transcription elongation, although it has also been found to function in all stages of transcription [4]. In yeast, cells lacking individual components of PAF1C exhibited decreased elongation efficiency [5,6,7]. Similarly, depletion of Paf1 in mouse myoblast cells caused the accumulation of Pol II on gene bodies and a slower Pol II accumulation rate, further verifying the positive role of PAF1C in elongation [8,9]. In vitro transcription assays showed that the human PAF1 complex could stimulate transcription elongation in a dose-dependent manner [10]. PAF1C upregulates gene expression through multiple mechanisms. As a Pol II-associated factor, PAF1C colocalizes with Pol II at actively transcribed genes [11,12]. Individual components of PAF1C can enhance the occupancy of PAF1C and Pol II on chromatin, although the recruitment mechanism is largely unclear. PAF1C can also interact with other transcription elongation factors through Pol II. Its interaction with SII can stimulate transcription in a synergistic manner [10]. In particular, due to the partially overlapping binding sites on Pol II, PAF1C can displace negative elongation factor (NELF) from the Pol II funnel to facilitate pause–release [13,14]. PAF1C can also regulate transcription epigenetically. It can promote histone H2B monoubiquitylation (at Lys123 in yeast or Lys120 in humans) through direct associations with the histone H2B-ubiquitylation deposition complex [15,16,17,18,19]. H2B ubiquitylation enhances the methylation of histone H3 at Lys4 and Lys79 [16,18,20,21], which eventually leads to elevated genome-wide gene expression.

PAF1C is a flexible protein complex, as most domains/regions of its components do not associate with one another to form a rigid structure. Therefore, it is very difficult to obtain an overall picture of the whole complex. Structural studies in recent years have significantly improved our understanding of the architecture of PAF1C. Crystal and cryo-electron microscopy (cryo-EM) structures of the multicomponent PAF1C from yeast [22,23], thermophilic fungi [24], and humans [13,25,26,27] revealed that Ctr9 and Paf1 form a rigid solenoidal core, while Cdc73, Rtf1 and Ski8 join the complex mainly through associations with Ctr9, the largest subunit. Of special note, Rtf1 shows varied binding affinities to PAF1C among species [3]. In yeast, Rtf1 tightly associates with PAF1C, while in other species, such as humans, RTF1 easily dissociates from PAF1C. Why Rtf1 behaves differently among species remains a mystery. Rtf1 can stimulate transcription elongation individually or as a component of PAF1C [25,28]. Rtf1 is required for H2B ubiquitylation [15,21,29,30,31], and therefore, it can also regulate transcription epigenetically. The mechanism by which Rtf1 stimulates H2B ubiquitylation is not fully understood. 

We previously solved the crystal structure of the ternary rigid core of PAF1C containing Ctr9, Paf1 and Cdc73 from a thermophilic fungus [24]. To understand how Rtf1 associates with the ternary complex, we reconstituted PAF1C from both budding yeast (*Saccharomyces cerevisiae*) and its close relative *Saccharomyces eubayanus*. We identified segments of Rtf1 in both species that were associated with the core of PAF1C in vitro and solved the crystal structure of the four-subunit core of PAF1C at high resolution. We observed detailed interactions among the components of the quaternary complex and found additional interacting surfaces between Rtf1 and Ctr9. We also analyzed the varied association features of Rtf1 with PAF1C from different species and found that the changes in both the C-terminal sequence of Rtf1 and the binding surfaces on Ctr9 among species could account for the varied binding affinities of Rtf1. Our work presents a precise structural model of the four-subunit core of PAF1C from a simple eukaryote, which may further facilitate our understanding of the function of PAF1C in vivo.

## 2. Results

### 2.1. In Vitro Reconstitution of the Quaternary PAF1C 

**Figure 1:**
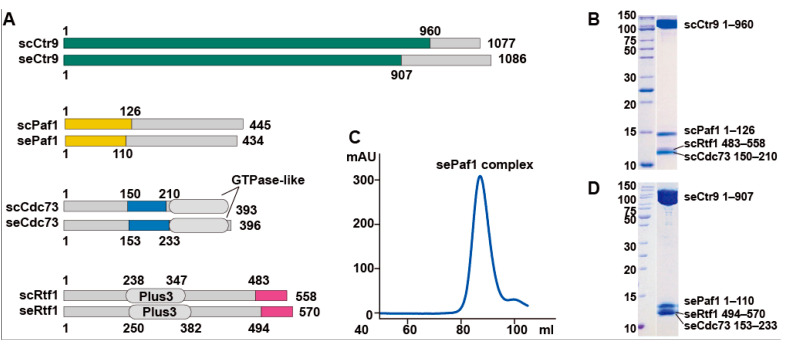
Reconstitution and purification of the quaternary PAF1C from two kinds of yeast. (**A**). Domain architecture of PAF1Cs from *Saccharomyces cerevisiae* (sc) and *Saccharomyces eubayanus* (se). Segments of Ctr9, Paf1, Cdc73 and Rtf1 used in structural studies are colored in green, yellow, blue and magenta, respectively. (**B**). Coomassie blue staining of PAF1C from *Saccharomyces cerevisiae*. (**C**,**D**). A gel-filtration profile (**C**) and the subsequent Coomassie blue staining (**D**) of the quaternary PAF1C from *Saccharomyces eubayanus*.

We previously solved the crystal structure of the ternary PAF1C composed of the core components of Ctr9, Paf1 and Cdc73 from a thermophilic fungus [24]. However, we were not able to add the Rtf1 component into the ternary complex to obtain a stable quaternary complex, as Rtf1 from the thermophilic fungus dissociated easily from the Ctr9-Paf1-Cdc73 ternary complex during further purification steps. For PAF1C from budding yeast, we could easily obtain a stable complex containing four components: Ctr9, Paf1, Cdc73 and Rtf1. Through serial deletions, we identified that the C-terminal fragment of Rtf1 containing residues 494 to 570 is the region responsible for forming a four-component PAF1C (Figure 1A). To understand the architecture of the four-component Ctr9-Paf1-Cdc73-Rtf1 complex, we first crystallized the complex from budding yeast (Figure 1B). As the crystals did not diffract well, we then used PAF1C from other species. We found that PAF1C from the yeast *Saccharomyces eubayanus*, which showed 86% sequence identity from that of budding yeast, could be purified and produce better crystals (Figure 1C,D). We then optimized the crystals of the complex of Ctr9-Paf1-Cdc73-Rtf1 from *Saccharomyces eubayanus* and solved the structure at the resolution of 2.7 Å (Supplementary Table S1).

### 2.2. Overall Structure of the Quaternary PAF1C

**Figure 2:**
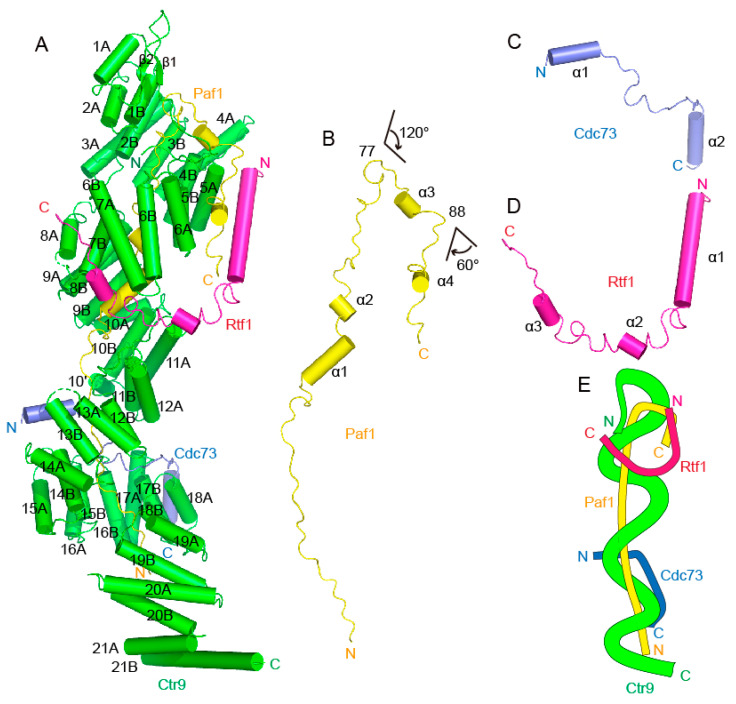
Overall structure of the quaternary PAF1C from *Saccharomyces eubayanus*. (**A**). A cartoon representation of the Ctr9-Paf1-Cdc73-Rtf1 four-component PAF1C. Ctr9 is colored green, Paf1 is colored yellow, Cdc73 is colored blue and Rtf1 is colored purple. (**B**–**D**). A cartoon representation of the single component Paf1 (**B**), Cdc73 (**C**) or Rtf1 (**D**) from the complex structure. (**E**). A simplified model showing the organization of the four-component PAF1C.

The overall architecture of the Ctr9-Paf1-Cdc73-Rtf1 quaternary complex from *Saccharomyces eubayanus* was shown to be similar as to those from the thermophilic fungus [24] and from humans [13] (Figure 2A). The largest component, Ctr9, forms the backbone and shapes the overall architecture of the complex, while the other components join the complex mainly through associations with Ctr9 (Figure 2A–D). Ctr9 folds into the shape of an elongated solenoid. The N-terminal region of Ctr9 contains two short β strands. The major body of Ctr9 folds into 21 tetratricopeptide repeats (TPRs) that wrap around the N-terminus of Paf1 to form a three-circled right-handed solenoid structure, with the Paf1 N-terminus just filling in the central tunnel of the Ctr9 solenoid. A typical TPR contains an A-helix and a B-helix connected by a loop. The TPR 10 of Ctr9 is atypical in that the loop connecting the two helices contains an additional short helix (labeled 10′) in the middle. Therefore, Ctr9 contains 43 helices in total. To facilitate crystallization, the C-terminal long α helix was truncated. As has been shown in the Ctr9 structures from other species, all TPRs of Ctr9 are organized into a two-layered wall-like structure, with the A-helices and the B-helices of the TPRs forming the outer layer and the inner layer of the solenoid surfaces (Figure 2A), respectively. Accompanying the spirally wound TPRs of Ctr9, a deep groove occurs between the edges of the spirally wound repeats (Figure 2A,E). The inner surface of Ctr9 interacts mainly with the N-terminus of Paf1, which is wrapped tightly inside the central tunnel of the Ctr9 solenoid and extends antiparallel to the direction that Ctr9 extends (Figure 2A,B). The rest of Paf1 extends out from the central tunnel and contacts the outer surface of Ctr9 (Figure 2A). Cdc73 and Rtf1 contact mainly the outer surface of Ctr9 (Figure 2C,D). Their interactions with Ctr9 were further strengthened by inserting a fragment into the groove of Ctr9 and making contacts with residues within the groove (Figure 2C,D). 

### 2.3. Structural Details of the Intermolecular Contacts between Paf1 and Ctr9

Paf1 residues 9–105 can be traced in the Ctr9-Paf1-Cdc73-Rtf1 quaternary complex. This segment of Paf1 has the shape of the letter “J”, which is mainly composed of unstructured loops, with only 4 short helices interspersed in the middle (Figure 2B). As this region of Paf1 does not fold into a structured domain, the shape of the letter “J” actually illustrates the track of Paf1 extending along the Ctr9 inner and outer surfaces. The N-terminal residues 16–76 of Paf1 that correspond to the linear part of the letter “J” are fully wrapped in the central tunnel of the Ctr9 solenoid. This region of Paf1 extensively contacts with the inner surface of Ctr9 (Figure 2A,B). At residue 77, Paf1 reaches the N-terminal end of the Ctr9 solenoid and then bends sharply by approximately 120 degrees, with residues 77–88 of Paf1 extending away from the Ctr9 inner tunnel and traversing across the edge of the two-layered TPRs of Ctr9 (Figure 2A,B). At residue 88, Paf1 reaches the outer surface of Ctr9 and bends again by approximately 60 degrees, which enables the remaining Paf1 residues (89–105) to extend along the outer surface of Ctr9 between helices 5A and 6A (Figure 2A,B). 

**Figure 3:**
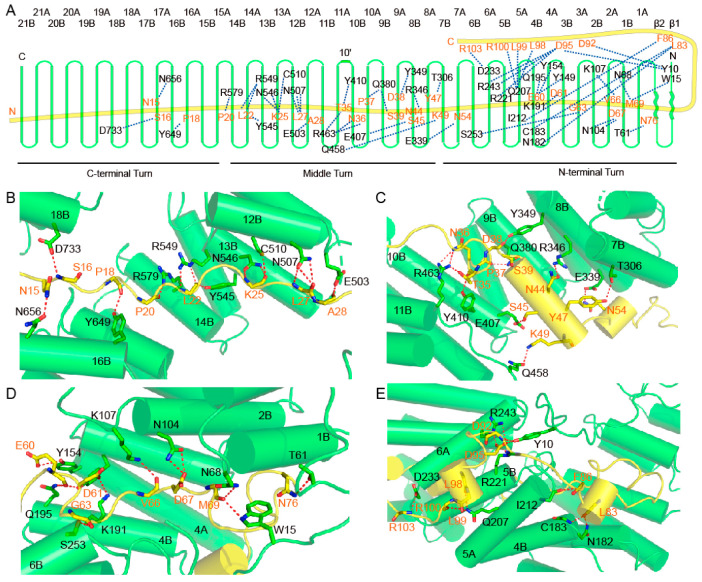
Molecular contacts between Ctr9 and Paf1. (**A**). A schematic representation of the hydrogen-bonding interactions between Ctr9 and Paf1. (**B**–**E**), Cartoon representations of the interacting details between Ctr9 and Paf1. Hydrogen bonds between residues are shown as red dots. Ctr9 and Paf1 are colored as in previous figures.

To facilitate analysis, the three circular turns of Ctr9 can be divided into the C-terminal, middle and N-terminal turns (Figure 3A). The inner surface of Ctr9 contains both hydrophobic and hydrophilic residues, similar to the inner buried Paf1, in which the hydrophobic and hydrophilic residues are interlaced. Therefore, both hydrophobic and hydrophilic interactions play important roles in stabilizing the contacts. The interactions between the very N-terminus of Paf1 and the C-terminal turn of Ctr9 are not very stable, as the electron densities of this region of both Paf1 and Ctr9 are poor. The middle turn of Ctr9 is in an extended conformation, as exemplified by a wider groove between the edges of the Ctr9 spiral (Figure 3B,C). A wider groove facilitates the exposure of the inner wrapped Paf1. The N-terminal turn of the Ctr9 spiral is much more compactly wound, resulting in a much narrower groove between the edges of the TPRs (Figure 3D). From the first traced N-terminal residue Ala9 to Pro33, approximately half the length of the inner buried Paf1 exhibits a much extended conformation (Figure 3A,B). The main chains of the residues Asn15, Ser16, Pro18, Pro20, Leu22, Lys25, Leu27 and Ala28 from Paf1 form one to three hydrogen bonds with the side chains of residues from the inner surface of Ctr9 (Figure 3A,B). From Pro33 to Asn76, Paf1 extends in a more twisted manner, as shown by the twisted loops and two α helices within this region. The twisted conformation enables Paf1 to contact more residues at the inner surface of Ctr9, so that more hydrogen bonds can be observed between Paf1 and Ctr9 within this segment. Residues Thr35, Asn36, Ser39, Asn44, Ser45, Tyr47, Lys49, Asn54, Glu60, Asp61, Asp67 and Asn76 of Paf1 form hydrogen bonds through their side chains with residues from Ctr9 (Figure 3A,C,D). Another fraction of residues, such as Pro37, Asp38, Glu60, Gly63, Val66 and Met69 of Paf1, forms hydrogen bonds through their main chains with the Ctr9 residues (Figure 3A,C,D).

From Lys77 to Asn88, Paf1 extends outside of the Ctr9 central tunnel and passes across the N-terminal edge of the Ctr9 solenoid (Figure 3E). This region of Paf1 interacts with Ctr9 mainly through hydrophobic interactions. Hydrogen bonds could also be observed between Leu83 and Phe86 of Paf1 and the residues Asn182, Cys183 and Ile212 that lie on the edge of the Ctr9 solenoid (Figure 3A,E). From residue Asn88, Paf1 turns its direction to extend along the outer surface of Ctr9 (Figure 3A). This segment of Paf1 (Val89 to Ser106) is sandwiched between helices 5A and 6A of Ctr9 (Figure 3E). The interaction between this segment of Paf1 and Ctr9 is also stabilized by both hydrophobic and hydrogen-bonding interactions. Asp92, Asp95, Leu98, Leu99, Arg100 and Arg103 from Paf1 form multiple hydrogen bonds with residues on the outer surface of Ctr9 (Figure 3A,E). Of note, Tyr10, which lies on the N-terminal loop of Ctr9, also forms hydrogen bonds with Asp92 and Asp95 from Paf1 (Figure 3A,E), indicating that the N-terminus of Ctr9 also plays a role in stabilizing the complex.

### 2.4. Structural Details of the Intermolecular Contacts between Cdc73 and Ctr9

**Figure 4:**
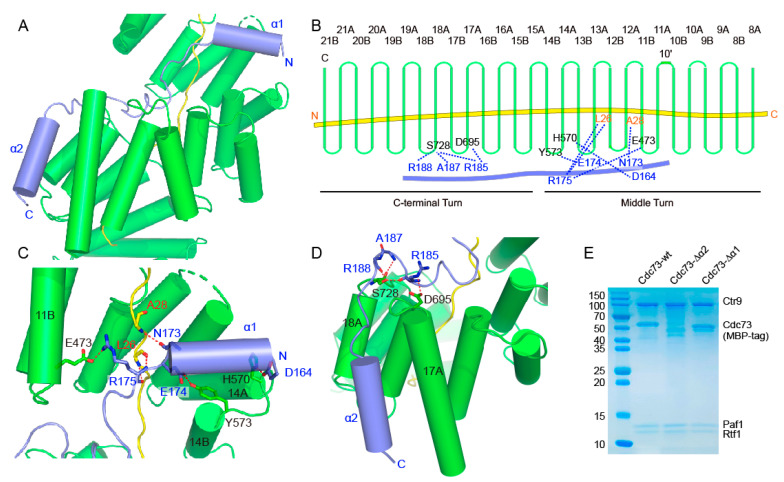
Molecular contacts between Ctr9 and Cdc73. (**A**). An overview of the interaction between Cdc73 and Ctr9. (**B**). A schematic representation of the hydrogen-bonding interactions between Cdc73 and Ctr9. Ctr9 and Cdc73 are colored as in previous figures. (**C**,**D**). Detailed hydrogen-bonding interactions between Cdc73 and Ctr9. (**E**). Truncations of Cdc73 to determine the contribution of each segment of Cdc73 to Ctr9 binding.

Cdc73 joins PAF1C mainly through its middle region, which contains two helices connected by loops, and contacts Ctr9 spanning a region containing the lower part of the middle turn and the upper part of the C-terminal turn (Figure 4A,B). The helix α1 of Cdc73 penetrates the groove of the Ctr9 solenoid from the edge between TPRs 13 and 14 (Figure 4C). This segment of Cdc73 almost floats on the edge of the TPRs. Hydrogen bonds can be observed between Asp164 of Cdc73 and His570 of Ctr9 and between Glu174 of Cdc73 and Tyr573 of Ctr9 (Figure 4B,C). At the C-terminal end of helix α1, Cdc73 reaches the inner-buried Paf1 (Figure 4D). As a result, Asn173 and Arg175 of Cdc73 form one and a pair of hydrogen bonds with Ala28 and Leu26 of Paf1, respectively (Figure 4B,D). Arg175 of Cdc73 also forms a salt bridge with Glu473 of Ctr9 (Figure 4B,D). From Arg175 to His180, Cdc73 extends alongside Paf1 within the groove of Ctr9, and then Cdc73 turns its direction and finds its path across the edge of TPRs to reach the outer surface of Ctr9 (Figure 4A,C). Reaching the outer surface of Ctr9, Cdc73 makes another turn to extend along the outer surface of Ctr9 between helices 17A and 18A (Figure 4A,D). Arg185, Ala187 and Arg188 are located at the turning point of Cdc73 and form several hydrogen bonds with Asp695 and Ser728 from Ctr9 to strengthen this interaction (Figure 4B,D). Threading out of the Ctr9 groove, Cdc73 mainly folds as helix α2 that extends in between and antiparallel to helices 17A and 18A (Figure 4D). The contacts at this region are also mainly mediated by hydrophobic interactions. To test the contribution of the structural elements of this region of Cdc73 to complex formation, we removed the N-terminal helix α1 or the C-terminal helix α2. Cdc73 without the α1 helix still formed a complex with the Ctr9-Paf1-Rtf1 ternary complex, while Cdc73 without α2 could no longer join the ternary complex (Figure 4E), indicating that the hydrophobic contacts by the C-terminal α2 are critical for Cdc73 to associate with Ctr9.

### 2.5. Structural Details of the Intermolecular Contacts between Rtf1 and Ctr9

**Figure 5:**
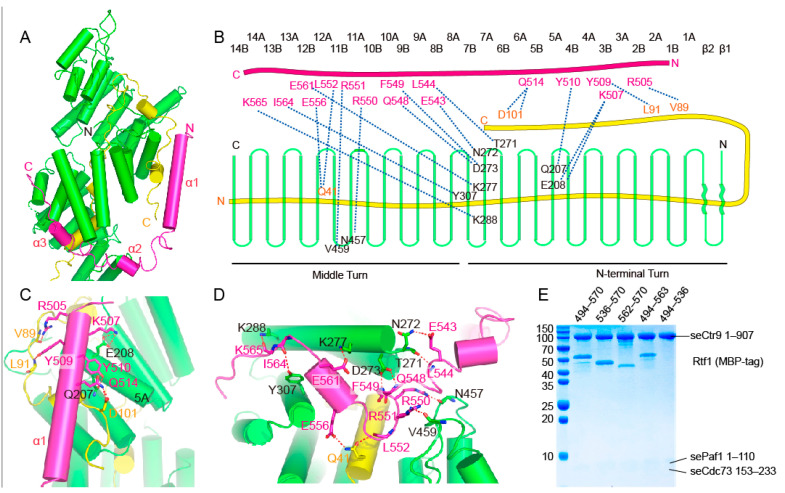
Molecular contacts between Rtf1 and Ctr9. (**A**). An overall representation of the interaction between Rtf1 and Ctr9. Ctr9 and Rtf1 are colored as in previous figures. (**B**). A schematic illustration of the interaction between Rtf1 and Ctr9. (**C**,**D**). Detailed hydrogen bonding interactions between Rtf1 and Ctr9. (**E**). Serial truncations to determine the contribution of each segment of Rtf1 to Ctr9 binding.

Yeast Rtf1 joins PAF1C through its C-terminal region. The overall structure of Rtf1 (residues 502 to 570) in the quaternary complex has the shape of a twisted letter “U” that contains three α helices connected by loops (Figure 5A). Rtf1 contacts Ctr9 spanning a region including the lower part of the N-terminal turn and the upper part of the middle turn of Ctr9 (Figure 5A,B). As Rtf1 and Cdc73 occupy different regions of Ctr9, they do not have direct interactions within this core structure. The long helix α1 of Rtf1 forms the right side of the letter “U” (Figure 5C). This part of Rtf1 contacts a surface composed of helix 5A of Ctr9 and the C-terminal segment of Paf1 that lies on the outer surface of Ctr9 (Figure 5C). In detail, Lys507 and Tyr510 of Rtf1 form hydrogen bonds with Glu208 and Gln207 of Ctr9, respectively, while Arg505, Tyr509 and Gln514 of Rtf1 form hydrogen bonds with Val89, Leu91 and Asp101 from Paf1, respectively (Figure 5B,C). Residues 531 to 552 of Rtf1 form the bottom of the letter “U” that lie in the groove of Ctr9 (Figure 5D). This side of Rtf1 makes extensive contacts with Ctr9. Residues Glu543, Leu544, Gln548 and Phe549 of Rtf1 form hydrogen bonds with residues Asn272, Thr271 and Asp273 from the edge on one side of the Ctr9 groove, while Arg550 and Arg551 of Rtf1 form hydrogen bonds with Asn457 and Val459 from the edge on the other side of the Ctr9 groove (Figure 5B,D). After residue Leu552, Rtf1 turns its direction to extend out of the Ctr9 groove, with the third α helix traversing across the edge of the Ctr9 wall, with the following loop of Rtf1 extending along the outer surface of Ctr9 between helices 7A and 8A (Figure 5D). This curved region of Rtf1 forms the left side of the letter “U”. Hydrogen-bonding interactions could be observed between Glu561, Ile564 and Lys565 of Rtf1 and Lys277, Tyr307 and Lys288 of Ctr9, respectively (Figure 5B,D). Hydrophobic interactions also play an important role in stabilizing complex formation, as observed by the clustered phenylalanine residues of the Rtf1 C-terminal tail and the hydrophobic surface of Ctr9. Of note, as Rtf1 extends across the central buried Paf1, interactions between Rtf1 and Paf1 could also be observed, with Leu552 and Glu556 of Rtf1 forming a hydrogen bond each with Gln41 of Paf1 (Figure 5B,D). 

To identify structural elements of Rtf1 responsible for association with the complex, we truncated Rtf1 by removing the long helix α1 or the C-terminal tail that binds the outer surface of Ctr9, or all the other parts except the C-terminal tail of Rtf1. We then mixed the Rtf1 fragments with the Ctr9-Paf1-Cdc73 ternary complex. Unexpectedly, we found that the long helix α1 of Rtf1 does not play a major role in complex formation, as its deletion did not noticeably change the binding of Rtf1 with the ternary complex (Figure 5E). In contrast, Rtf1 without the C-terminal tail or with only the C-terminal tail showed reduced binding to the ternary complex (Figure 5E), indicating that the very C-terminus and the segment that binds within the groove of Ctr9 are two key segments important for Rtf1 to associate with PAF1C.

### 2.6. Different Binding Modes of the Rtf1 C-Terminus to PAF1C

**Figure 6:**
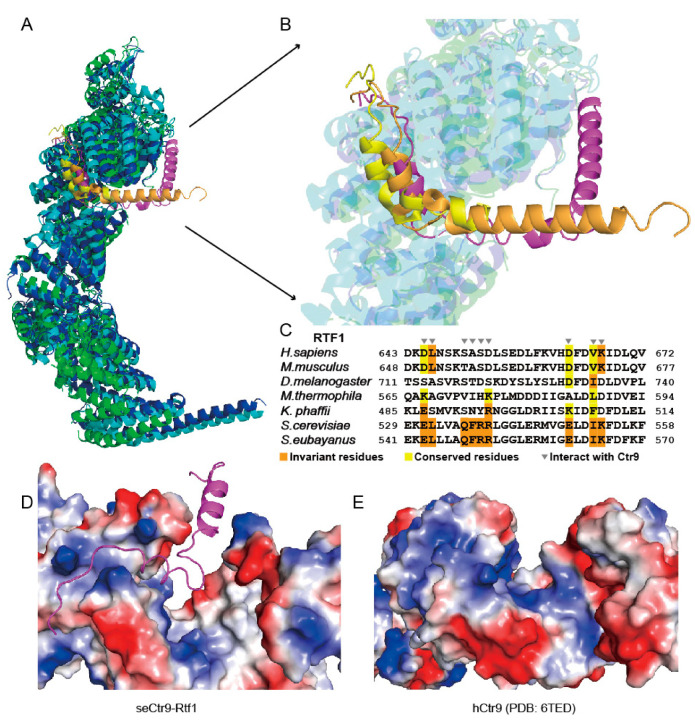
Sequence and structure analysis of the interacting residues and surfaces between Rtf1 and Ctr9. (**A**). Superimposition of the core structures of the Paf1 complex from *Saccharomyces eubayanus* (this study), *Saccharomyces cerevisiae* (PDB: 7DKH) and *Komagataella phaffii* (PDB: 7XN7). (**B**). A closeup view of the Rtf1-Ctr9 interacting regions in A. (**C**). Sequence alignment of the C-terminal regions of Rtf1 from different species. (**D**,**E**). Electrostatic representation of the Rtf1 binding surface on Ctr9 in *Saccharomyces eubayanus* (**D**), this study) and its corresponding surface in humans (**E**), PDB: 6TED).

A similar region of Rtf1 in budding yeast has been solved recently [22]. The binding mode of Rtf1 to PAF1C shown in this study is slightly different from that shown in previous studies [22,23] (Figure 6A,B). The left side and the bottom of the “U”-shaped Rtf1 in this study superimposed well with the corresponding regions in previous studies [22,23] (Figure 6A,B). The α1 helix that corresponds to the right side of the “U”-shaped Rtf1 in this study showed a completely different conformation from that shown in previous studies (Figure 6A,B). This corresponding region of Rtf1 in previous studies either was not visible [23], or was directed in a different direction [22], which may be due to crystal packing, as it interacts with another molecule in the crystal. In our study, we observed that helix α1 of Rtf1 actually interacts with a new surface made of Ctr9 and Paf1. With this additional binding surface, the total contact surfaces between Rtf1 and the PAF1C core subunits from *Saccharomyces eubayanus* reached to 3025 Å^2^, which is much larger than the corresponding contact surfaces from *Saccharomyces cerevisiae* or *Komagataella phaffii*, which are 1754 Å^2^ or 1509 Å^2^, respectively. This interaction may facilitate the association between Rtf1 and Ctr9, complementing the missing information on the role of Rtf1 in complex formation. However, our further work showed that the contribution of helix α1 of Rtf1 to complex formation is minimal, as its deletion did not notably change quaternary complex formation (Figure 5E). This indicates that helix α1 of Rtf1 may have several conformations during complex formation. PAF1C structures shown in this study and in the previous studies [22,23] each captured a different conformation of Rtf1. The functional significance of this segment of Rtf1 is not clear.

As has been shown previously [3], Rtf1 showed varied binding behaviors to join PAF1C among different species. Sequence alignment with the C-terminal regions of Rtf1 from different species showed that the sequences changed dramatically, especially those Ctr9-binding residues (Figure 6C). In yeast, such as in *Saccharomyces cerevisiae*, *Saccharomyces eubayanus* (this study) and *Komagataella phaffii*, Rtf1 associates tightly with PAF1C. We previously found that Rtf1 in the thermophilic fungus also dissociated easily from PAF1C during further purification steps [24]. We also reconstituted the human PAF1C and found that RTF1 dissociated easily from PAF1C. We then tested the addition of the Rtf1 C-terminus from *Saccharomyces eubayanus* to PAF1Cs from other species. We found that the Rtf1 C-terminus from *Saccharomyces eubayanus* could not associate with PAF1C from either the thermophilic fungus or humans (Supplementary Figure S1). We compared the Rtf1 binding surface in *Saccharomyces eubayanus* and the corresponding surface of CTR9 in humans, and found that the electrostatic distributions of both surfaces are quite different, indicating that the changed Rtf1-binding surfaces of Ctr9 from thermophilic fungi and from humans (Figure 6D,E) may also contribute to their loss of binding to the Rtf1 C-terminus.

## 3. Discussion

PAF1C is a eukaryote-specific transcription elongation factor that functions mainly as a positive regulator that upregulates genome-wide gene transcription. To date, considerable structural progress has been made on PAF1C from different species, which has dramatically improved our understanding of its molecular mechanisms of transcriptional regulation and its association with RNA polymerase II. However, due to the dynamic features of PAF1C, a complete precise model of PAF1C is still lacking. In this study, we solved a high-resolution quaternary PAF1C of Ctr9-Paf1-Cdc73-Rtf1 from *Saccharomyces eubayanus*. This structure can be considered a complete model of the rigid core of PAF1C from a lower monocellular eukaryote. As the unseen domains or regions of PAF1C do not associate with the quaternary core, the structures of those domains could only be visualized when stabilized on the Pol II-containing complexes. 

By solving the Ctr9-Paf1-Cdc73-Rtf1 complex structure, we established a high-resolution model of the quaternary complex and found that the overall structure of the rigid core of PAF1C from different species is quite similar. Our study further proved that Ctr9 and Paf1 are two founding members of PAF1C. The solenoidal structure composed of the Paf1 N-terminus and the spirally wound Ctr9 shapes the overall architecture of the rigid core of PAF1C. As the binary complex can be stably isolated in vitro, and loss of either resulted in the most deleterious effects in vivo [3], the Ctr9-Paf1 binary complex could be the smallest functional unit of the whole complex. The Ctr9-Paf1 binary complex, especially the Ctr9 component, functions as a platform that is responsible for the recruitment of all the other components of PAF1C. Cdc73 binds Ctr9 through its middle region, while Rtf1 binds Ctr9 through its C-terminus. Both proteins contact the outer surface of Ctr9 mainly through hydrophobic interactions. In addition, both proteins strengthen their associations with Ctr9 by inserting a segment into the surface groove of Ctr9. After joining PAF1C, both proteins also have contacts with Paf1, although the contacts may not contribute much to complex formation. The sixth component, Ski8, in the human PAF1C also joined the complex by interacting with the outer surface at the bottom region of Ctr9. Of special note, Rtf1, Cdc73 and Ski8 occupy the top, middle and bottom of the elongated solenoidal Ctr9, respectively. These three proteins do not have direct contacts with one another on the solenoidal core, indicating that these three components can be recruited to PAF1C individually. Our study presents a model of the interacting components of the Ctr9-Paf1-Cdc73-Rtf1 quaternary core. The quaternary core does not contain segments from Leo1, as Leo1 is not associated with the quaternary core. Leo1 is associated with the region of Paf1 that lies outside of the solenoidal structural core [27] and functions as an extended arm to hold Pol II [13,25].

During the structure resolution, we found that the electron density of the N-terminal half of Ctr9 was well defined, exemplified as clear densities for the side chains of most residues. For the C-terminal half of Ctr9, starting from TPR 12 until the C-terminal end, the electron density was poor. To verify the structural model, five predicted models of Ctr9 by Alphafold2 [32] server were generated as references. However, when the N-terminal halves of the alphafold2-derived models were superimposed, their C-terminal halves showed large structural deviations (Supplementary Figure S2). The C-terminal half of our experimental determined model of Ctr9 showed even larger deviations from those of the predicted models when their N-terminal halves were superimposed (Supplementary Figure S2). Similarly, the C-terminal half of our model of PAF1C deviated from the corresponding region of PAF1C from budding yeast by an angle of around 17° when their N-terminal halves were superimposed (Supplementary Figure S2). This indicates that the elongated core of PAF1C has some degree of flexibility, which may facilitate the Paf1 complex in adopting a suitable conformation to associate with Pol II. 

Components of PAF1C change their sequences and lengths dramatically during evolution. For example, the molecular weight of the human PAF1C is approximately 1.3-fold larger than that of the yeast PAF1C. Despite these changes, the overall architecture of PAF1C and the molecular contacts among the components are largely conserved, with Rtf1 as an exception. In budding yeast and its highly homologous Pichia yeast, Rtf1 associates tightly with Ctr9 through its C-terminus. In higher eukaryotes, or even in another simple eukaryote, the thermophilic fungus, Rtf1 changes its manner of association with PAF1C. The C-terminal regions of Rtf1 in the thermophilic fungus and in humans are no longer responsible for association with PAF1C. Therefore, how and why Rtf1 changed its interaction with PAF1C during evolution remains a mystery. In this study, we found that Rtf1 interacts with both Ctr9 and Paf1 through its C-terminal region, with the Rtf1-Ctr9 interaction playing a dominant role in complex formation. However, during evolution, the sequence of the C-terminal Rtf1 changed dramatically. Concomitantly, its binding surface on Ctr9 also changed. Cryo-EM studies of PAF1C in complex with Pol II revealed that Rtf1 from both humans and Pichiaceae yeast contacts the Paf1-Leo1 dimerization domain on the Pol II surface. The binding to Ctr9 through its C-terminus could only be observed on Rtf1 from yeast but not from humans. Therefore, the function of the C-terminus of RTF1 in higher eukaryotes and why the Rtf1 C-terminus loses its binding to PAF1C during evolution remain elusive. During evolution, Rtf1 increased in molecular weight and developed more functions. Therefore, it is reasonable that a weaker association with PAF1C may facilitate its individual function independent of PAF1C. 

## 4. Materials and Methods

### 4.1. Protein Expression and Purification

The open reading frames of Ctr9 (Uniprot: A0A0L8RHL9), Paf1 (Uniprot: A0A0L8RM45), Cdc73 (Uniprot: A0A0L8RF82) and Rtf1 (Uniprot: A0A0L8RIY1) from *Saccharomyces eubayanus* were chemically synthesized with codon optimization for efficient bacterial expression. Subunits of PAF1C from *Saccharomyces eubayanus* were subcloned into two compatible vectors for coexpression in *E.coli*. Ctr9(1–907) without any tag and SUMO-tagged Paf1(1–110) were cloned into a pCDFDuet-1 vector. His-SUMO-tagged Rtf1(494–570) and Cdc73(153–233) without any tag were cloned into a RSFDuet-1 vector. These two plasmids were co-expressed in *E.coli* strain BL21(DE3) at 37 °C till the OD_600_ reached around 1.0. Then, after cooling the cells at 20 °C for around an hour, 0.2 mM IPTG was added to induce expression overnight. Cells were harvested via centrifugation at 5000 rpm for 10 min. Cell pellets were resuspended with the buffer containing 20 mM Tris-pH 8.0, 200 mM NaCl and 20 mM imidazole and sonicated for 5 min. The supernatant was fractionated via centrifugation of the cell lysate at 18,000 rpm for an hour. Histidine-SUMO-tagged target protein was isolated through a nickel-charged HiTrap Chelating FF column from GE healthcare. Both the SUMO and the histidine-SUMO tags were then cleaved by incubating with the histidine-tagged ULP1 protease. The samples were then loaded directly onto a heparin column to remove bound DNA. Target protein was separated by increasing the salt concentration of the low salt buffer (20 mM Tris-pH 7.0, 200 mM NaCl, 2 mM DTT) from 200 mM to 1 M NaCl through a linear gradient. Finally, the eluted target protein was further purified on a HiLoad 200 16/600 gel-filtration column. After these purification steps, the target protein was of high purity and homogeneity. The purified protein was concentrated to around 16 mg/mL and stored in a −80 °C freezer.

cDNA clones encoding full-length human PAF1C subunits were chemically synthesized. cDNAs of CTR9 (Uniprot: Q6PD62), PAF1 (Uniprot: Q8N7H5), LEO1 (Uniprot: Q8WVC0), CDC73 (Uniprot: Q6P1J9) and SKI8 (Uniprot: Q9GZS3) were used as PCR templates for insertion into the 438-C (Addgene, 55220) and 438-A (Addgene, 55218) vectors via ligation-independent cloning [33]. All subunits were incorporated into a single plasmid by performing successive rounds of ligation-independent cloning. The template of RTF1 (Uniprot: Q92541) was inserted into a pFastBac1 plasmid and was expressed separately.

Bacmid, virus and protein production were conducted following a standard Bac-to-Bac baculovirus expression protocol. Sf9 cells infected by viruses were collected via centrifugation (1500× *g*, 4 °C, 20 min), resuspended in lysis buffer (20 mM Tris pH 7.5, 200 mM NaCl, 20 mM imidazole pH 7.5 and 1 mM PMSF) at 4 °C and then sonicated for around 5 min. The soluble fraction of the cells was collected via centrifugation of the cell lysate at 18,000 rpm for 1 h. His-MBP-TEV-tagged human PAF1C was isolated through a nickel-charged HiTrap Chelating FF column. The His-MBP-TEV tag was cleaved using a TEV protease and then dialyzed with the initial buffer at 4 °C overnight. The dialyzed solution was reloaded onto a nickel-charged chelating column to remove both His-MBP-TEV tag and the TEV protease. The flow-through was concentrated and purified through a HiLoad 200 16/600 gel filtration column with the buffer containing 20 mM HEPES at pH 7.5, 100 mM NaCl and 2 mM DTT. Peak fractions were analyzed using SDS–PAGE. Purified proteins were concentrated to around 20 mg/mL and stored in a −80°C freezer.

### 4.2. Crystallization and Structure Determination

Crystallization was carried out using the hanging-drop, vapor-diffusion method by mixing equal volume of protein and well solution. Crystals of the quaternary complex containing Ctr9(1–907), Paf1(1–110), Rtf1(494–570) and Cdc73(153–233) from *Saccharomyces eubayanus* were grown at 20 °C by mixing 0.5 µL protein at the concentration of 16 mg/mL with 0.5 µL crystallization buffer containing 0.2 M Ammonium citrate tribasic pH 7.0 and 25% *w*/*v* PEG 3350.

Data sets for the crystals of the native proteins were collected at the Shanghai Synchrotron Radiation Facility (SSRF) beamline BL17U1 in China. Data sets for crystals of the selenomethionine-labeled wild-type samples were collected at SSRF beamline BL18U1 at the wavelength of 0.97918 Å. All the data sets were collected at the temperature of 100 K. All the data sets were processed with the program HKL2000 (Version 716) [34]. Structure determination was carried out with PHENIX (Version1.13-2998) [35] using the data sets of the selenomethionine-labeled crystals of the wild-type samples through the SAD method. Of 23 selenium atoms, 19 were identified, which were used to solve the initial phase with a partial model. The partial model was manually rebuilt by Coot (Version 0.8.7) [36], and further refined using PHENIX. After an almost complete model was built, the selenomethionine-labeled model was used as the starting model for the native data set for further refinement. As the native data set showed a high degree of anisotropy, the STARANISO server (https://staraniso.globalphasing.org/cgi-bin/staraniso.cgi, accessed on 6 May 2023) was used to re-process the data. The resultant data set generated better electron density maps, which facilitated the model building process. The structures of the native and the selenomethionine-labeled PAF1Cs are quite similar, except that more residues were built in the model of the native complex. The root-mean-square-deviation (RMSD) between 1054 aligned residues from both models is 0.918 Å.

### 4.3. Gel Filtration Analysis

The ternary complex of Ctr9(1–907)-Paf1(1–110)-Cdc73(153–233) from *Saccharomyces eubayanus* was obtained through a coexpression method as described above. The His-MBP-tagged Rtf1 fragments were purified for better staining. The purified Ctr9-Paf1-Cdc73 ternary complex and His-MBP-tagged Rtf1 were mixed in a 1:7 ratio on ice for half an hour; then, the mixture was loaded onto a Superdex 200 column. Complex fractions were pooled and concentrated for further analysis.

Th ternary complex of Ctr9(1–907)-Paf1(1–110)-Rtf1(494–570) and His-MBP-tagged Cdc73 fragments (153–233, 174–233, 153–190) from *Saccharomyces eubayanus* were purified separately. The purified Ctr9-Paf1-Rtf1 and His-MBP-tagged Cdc73 were mixed in a 1:5 ratio on ice for half an hour; then, the mixture was loaded onto a Superdex 200 column. Complex fractions were pooled and concentrated for further analysis.

### 4.4. Related Chemicals

Ammonium citrate tribasic (Sigma-Aldrich A1332, St. Louis, MO, USA, ≥97%); PEG3350 (Hampton HR2-591, Aliso Viejo, CA, USA); Tris (VWR CHLC0497, Radnor, PA, USA, Ultra-Pure Grade); Sodium Chloride (VWR C0241, ≥99.9%); DTT (Sigma-Aldrich D0632, ≥99%); IPTG (BBI A600168, Shanghai, China, ≥99%); HEPES (Sigma-Aldrich V900477, ≥99.5%); Imidazole (Biotopped I6090, Beijing, China, ≥98%).

## Data Availability

The coordinates of the native and the selenomethionine-labeled PAF1C were deposited in the protein data bank (https://www.rcsb.org, accessed on 8 May 2023) with the accession numbers 8J8P and 8J8Q, respectively.

## Supplementary Materials

The following supporting information can be downloaded at: https://www.mdpi.com/article/10.3390/ijms24108730/s1, Supplementary materials contain Supplementary Figures S1, S2 and Supplementary Table S1.
