# Supplementary material for: Structural Basis of the Transcriptional Elongation Factor Paf1 Core Complex from Saccharomyces eubayanus
Source: Int J Mol Sci. 2023 May 13;24(10):8730. doi: 10.3390/ijms24108730 (PMC10217977; doi:10.3390/ijms24108730)
Supplement: Supplementary file 1 [file ijms-24-08730-s001.zip › ijms-2318932-supplementary.pdf]

Crystal	Native Ctr9-Paf1-Cdc73-Rtf1	SeMET-labeled Ctr9-Paf1-Cdc73-Rtf1
PDB Code	8J8P	8J8Q
Beam line	SSRF-BL17U1	SSRF-BL18U1
Wavelength	0.97918	0.97930
Space group	$P2_1$	$P2_1$
Unit cell		
a, b, c (Å)	71.8, 88.5, 127.8	71.6, 89.2, 128.1
α, β, γ (°)	90.0, 97.3, 90.0	90.1, 97.1, 90.0
Resolution (Å)	50.0–2.70 (2.75–2.70)[a]	50.0–3.10 (3.15–3.10)
R_{sym}	0.068 (0.706)	0.157 (1.116)
I/σ (I)	22.1 (1.6)	12.9 (1.3)
Completeness (%)	99.9 (100)	99.5 (99.1)
Redundancy	3.7 (3.6)	6.7 (5.7)
Number of unique reflections	43763	28757
R_{work}/R_{free} (%)	22.5/26.3	23.6/27.9
Number of none-H atoms		
Ctr9	7363	7031
Paf1	726	784
Cdc73	366	347
Rtf1	594	594
H2O	79	/
Average B factors (Å^2)		
Ctr9	96.5	77.1
Paf1	50.3	37.6
Cdc73	109.8	87.5
Rtf1	68.2	47.0
H2O	47.0	/
R.m.s. deviations		
Bond lengths (Å)	0.008	0.009
Bond angles (°)	1.034	1.037
Ramachandran plot		
Favored (%)	93.3	94.91
Allowed (%)	6.7	5.09
Disallowed (%)	0	0
MolProbity score	2.42	3.24

[a] Highest resolution shell (in Å) shown in parentheses.

Supplementary Table S1. Data collection and refinement statistics.

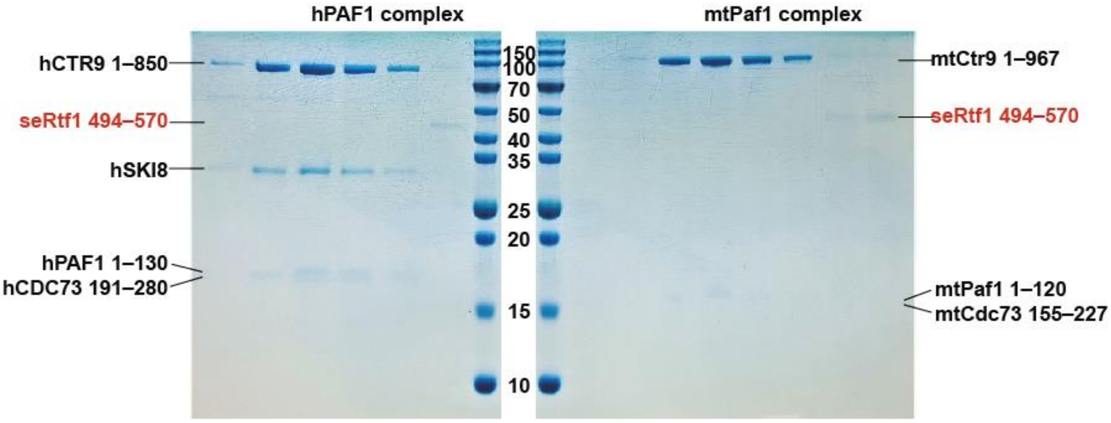

Supplementary Figure S1. Yeast Rtf1 C-terminus does not associate with the Paf1 complex from higher eukaryotes. Gel-filtration analysis showed that the C-terminus of Rtf1 from *Saccharomyces eubayanus* (se) did not bind the PAF1 complex from humans (h, left), nor did it bind the Paf1 complex from the thermophilic fungus *Myceliophthora thermophile* (mt, right).

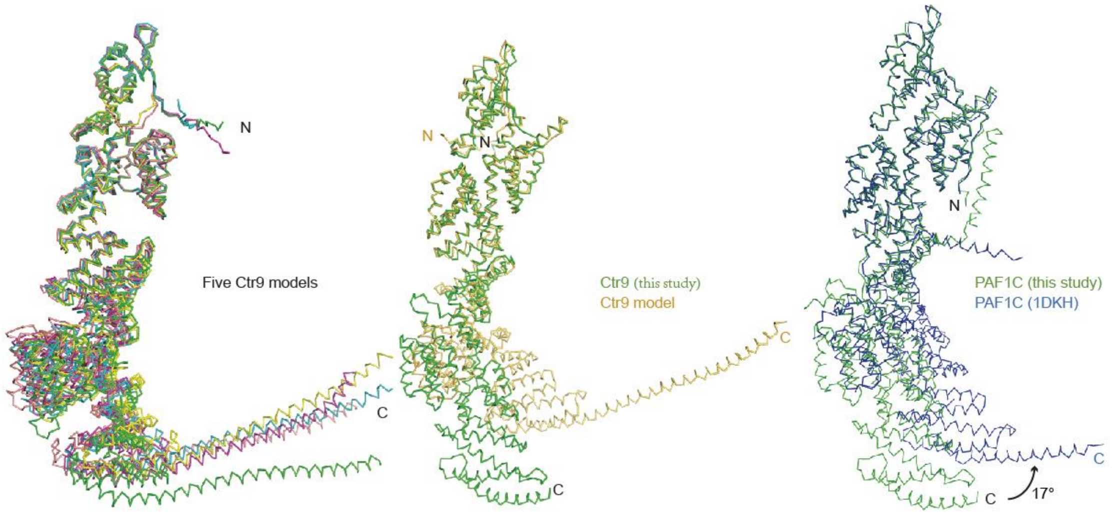

Supplementary Figure S2. Structural alignments of different Ctr9 models. Left, Five Alphafold2-prediced models of Ctr9 from *Saccharomyces eubayanus* were aligned. The models were shown in ribbon representations and were colored yellow, red, blue, green and cyan, respectively. Middle, Crystallography-solved structure of Ctr9 (green, this study) and a Alphafold2-predicted Ctr9 model (yellow) from *Saccharomyces eubayanus* were aligned. Right, The N-terminal halves of the PAF1C structures from *Saccharomyces eubayanu* (this study, residues 10-490) and budding yeast (1DKH) were aligned, with a RMSD of 1.36 Å between aligned residues.
